# Sex differences in rTMS treatment response: A deep learning‐based EEG investigation

**DOI:** 10.1002/brb3.2696

**Published:** 2022-07-25

**Authors:** M. Adamson, A. L. Hadipour, C. Uyulan, T. Erguzel, O. Cerezci, R. Kazemi, A. Phillips, S. Seenivasan, S. Shah, N. Tarhan

**Affiliations:** ^1^ Department of Neurosurgery Stanford University School of Medicine Stanford California; ^2^ Department of Rehabilitation Service VA Palo Alto Healthcare System Palo Alto California; ^3^ Department of Cognitive Sciences University of Messina Messina Italy; ^4^ Department of Mechanical Engineering İzmir Katip Çelebi University İzmir Turkey; ^5^ Faculty of Engineering and Natural Sciences Üsküdar University Istanbul Turkey; ^6^ Faculty of Health Sciences Üsküdar University Istanbul Turkey; ^7^ Department of Cognitive Psychology Institute for Cognitive Science Studies Tehran Iran; ^8^ Faculty of Humanities and Social Sciences Üsküdar University Istanbul Turkey

**Keywords:** EEG, Deep Learning, rTMS, depression, Sex Differences, Iran

## Abstract

**Introduction:**

The present study aimed to investigate sex differences in response to repetitive transcranial magnetic stimulation (rTMS) in Major Depressive Disorder (MDD) patients. Identifying the factors that mediate treatment response to rTMS in MDD patients can guide clinicians to administer more appropriate, reliable, and personalized interventions.

**Methods:**

In this paper, we developed a novel pipeline based on convolutional LSTM‐based deep learning (DL) to classify 25 female and 25 male patients based on their rTMS treatment response.

**Results:**

Five different classification models were generated, namely pre‐/post‐rTMS female (model 1), pre‐/post‐rTMS male (model 2), pre‐rTMS female responder versus pre‐rTMS female nonresponders (model 3), pre‐rTMS male responder vs. pre‐rTMS male nonresponder (model 4), and pre‐rTMS responder versus nonresponder of both sexes (model 5), achieving 93.3%, 98%, 95.2%, 99.2%, and 96.6% overall test accuracy, respectively.

**Conclusion:**

These results indicate the potential of our approach to be used as a response predictor especially regarding sex‐specific antidepressant effects of rTMS in MDD patients.

## Graphical abstract

Maheen Adamson, Srija Seenivasan, Caglar Uyulan, Turker Erguzel, Onder Cerezci, Reza Kazemi, Siddhi Shah, Angela Phillips, Nevzat Tarhan. Sex differences in rTMS treatment response: A deep learning‐based EEG investigation. We developed a novel pipeline based on convolutional LSTM‐based deep learning to classify 25 female and 25 male patients based on their rTMS treatment response. Five different classification models were generated: pre‐/post‐rTMS female (model 1), pre‐/post‐rTMS male (model 2), pre‐rTMS female responder /nonresponder (model 3), pre‐rTMS male responder/nonresponder (model 4), and pre‐rTMS responder/nonresponder (model 5), achieving respectively 93.3%, 98%, 95.2%, 99.2%, and 96.6% overall test accuracy, and indicating the potential of our approach to be used as a response predictor especially regarding sex‐specific antidepressant effects of rTMS in MDD patients. These results indicate the potential of our approach to be used as a response predictor of sex‐specific antidepressant effects of rTMS in MDD patients and to help guide clinicians to administer more personalized interventions.

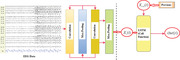

## 1 INTRODUCTION

Major depressive disorder (MDD) is a psychiatric condition concomitant of dysregulated mood and one of the leading causes of disability worldwide, affecting approximately 17% of the population (Kessler et al., 2003; Picco et al., 2017; Seney et al., 2018). Prevalence of MDD has been estimated to be twice as high in women, and females typically have more severe and a greater risk of recurrent episodes than men (Perugi et al., 1990). There is evidence regarding the role of sex and reproductive hormones in the pathophysiology of depression, as well as differences in the brain at the structural, cellular, and network levels that can potentially contribute to the susceptibility of sex to MDD (Tripp et al., 2012). Sex hormones have been shown to modulate functional neural networks, such as modulation of the central glutaminergic and serotonergic system by ovarian steroids. Considering the evidence regarding sex‐dependent physiological and neural dynamics, it is possible to form hypotheses regarding potential differences between men and women, in terms of response to treatment across different conditions and treatments (Huang et al., 2008; Rubinow & Schmidt, 2019). Investigation of the potential differences between men and women has been the focus of the current research, with the condition and treatment of focus being MDD and repetitive transcranial magnetic stimulation (rTMS), respectively.

Repetitive TMS is a noninvasive, nonpharmacological brain stimulation treatment that takes advantage of a pulsed magnetic field near the scalp to induce neuronal depolarization in a targeted brain region. TMS for MDD starts with motor threshold (MT) determination, which calibrates the stimulator to an individual's cortical excitability. During MT determination, a clinician delivers single pulse TMS to the motor cortex, and records the amount of stimulator output necessary to induce movement in the contralateral hand in 50% of delivered pulses. Following calibration, a course of TMS is delivered to the prefrontal cortex at 120% of MT on a daily basis for up to 30 (or more) sessions, often followed by a taper phase (Philip et al., 2018). TMS treatment protocols have shown preliminary clinical efficacy for numerous neuropsychiatric and behavioral disorders; randomized clinical trials recently led to the US FDA approvals for TMS to treat OCD and for smoking cessation (Greenberg et al., 2021). The dorsolateral prefrontal cortex (DLPFC) of the brain is shown to be correlated with the cognitive‐control and affective network thus playing a significant role in mood regulation (Downar & Daskalakis, 2013). Repetitive transcranial magnetic stimulation of the left DLPFC has been shown to induce an inverse correlation between resting connectivity of the DLPFC and the default mode network (DMN), a dysfunctional circuit characteristic of MDD (Williams et al., 2021). Superior clinical outcomes were associated with targets exhibiting the greatest DLPFC‐to‐sgACC negative connectivity, or anticorrelation, suggesting the antidepressant mechanism of TMS (Philip et al., 2018). TMS uses a pulsed magnetic field to induce neuronal depolarization in a targeted brain region, either “high” (≥5 Hz) or “low” (≤1 Hz), considered excitatory and inhibitory, respectively (Philip et al., 2018).

Sexual dimorphism has been observed in MDD patients not only in lifetime risk, clinical presentation, but also in their response to pharmacotherapy, perhaps attributed to sex‐specific biological differences, including hormone levels and metabolic enzymes, which all together lead to different profiles of pharmacokinetics and pharmacodynamics (Hu et al., 2021). Interestingly, the neural networks that include the DLPFC have been previously shown to be affected by ovarian sex hormones that may suggest the possibility of sex‐dependent rTMS treatment outcome (Huang et al., 2008; Rogers & Dhaher, 2017). Based on our recent work, it is apparent that sex, especially in the context of depression and rTMS treatment outcome, has not received appropriate research attention (Phillips et al., 2020). Therefore, this study was designed to investigate the sex differences in response to neuromodulation in major depressive disorder using state of the art deep learning methods applied on electroencephalography (EEG) data.

Among the numerous modalities of neuroimaging, EEG is by far the least expensive and complicated which makes it a popular neuroimaging method especially in clinical settings (Zhang et al., 2021b). With its high temporal resolution and noninvasive characteristics, the EEG measurements of the sum of synaptic potentials, changes in the amplitude, and latency of cortical reactivity can be reflected in the corticocortical interactions on a millisecond time‐scale (de Aguiar Neto & Rosa, 2019; de la Salle et al., 2020). EEG can measure cordance, a computation of regional brain activity, that has a strong correlation with cerebral brain perfusion, and has been used in the past before and after TMS to assess cortical inhibition and excitation, connectivity, and pharmacology of TMS in patients with MDD (Leuchter et al., 2009; Tremblay et al., 2019). With the use of EEG data, one can compute its recordings of band power, alpha asymmetry, signal‐based features, network‐based features, or evoked potentials to characterize the brains’ functional response (Bares et al., 2015).

EEG, among many other types of data, can be used after artifacts are removed, in deep learning models for the purposes of feature extraction and thus classification of a variable of interest (Abbasi & Goldenholz, 2019; Ay et al., 2019; Craik et al., 2019). Working with the raw (uncleaned) data is not preferable in the modeling via deep learning. Because the learning process interferes with the noise, it may cause overfitting or biased learning. “Raw” term is generally stated as clean data after preprocessing (noise filtered). Deep learning automatically extracts valuable features from the preprocessed data. The artifacts originated from various physiological or nonphysiological sources such as eye movements, blinks, and cardiac or muscle activity, blood pressure, and magnetic field of electronic devices, mobiles wave, power‐line, while continuous EEG recordings are collected. The artifact‐corrected EEG can be estimated by subtracting the pure artifact activity, which is calculated as the product of modeled artifact patterns from the original artifact‐contaminated EEG.

Convolutional neural networks (CNN), as a popular deep learning model, has been used for biosignal classification in the past (Chen et al., 2020; Nguyen et al., 2020; Ouhame et al., 2021; Zhang et al., 2021a). LSTM was developed for overcoming the challenge to address long‐term information preservation and short‐term input skipping in latent variable models. LSTM inherits a memory cell that has the same shape as a hidden state. The memory cell is controlled by several gates (i.e., input, output, forget). These gates represent a dedicated mechanism, which can decide when to remember and when to ignore inputs in the hidden state. The combination of CNN and LSTM is designed for sequence prediction problems with spatial inputs, like images or videos. The combination means that CNN‐LSTM architecture involves using CNN layers for feature extraction on input data combined with LSTMs to support sequence prediction. Therefore, there is no need for any feature extraction process before training the deep learning architecture (Abdelhameed et al., 2018; Courtney & Sreenivas, 2020; Nagabushanam et al., 2020). Improved and recurrent neural networks (RNN) both perform optimally when classifying EEG data, but due to size and type of data set, LSTM has been used for optimal results here. Offering a high predictive value, machine learning approaches have been widely used in psychiatry research (Yao et al., 2020). Deep learning algorithms for the purposes of classifying psychiatric illnesses and predicting treatment outcomes have yielded successful results in some recent studies (Cosmo et al., 2021; de Bardeci et al., 2021; Etkin, 2019; Zhang et al., 2021a). Recently, taking advantage of signal processing methods in the context of mood disorders has been extensively studied, however, implementing them is challenging due to disorder heterogeneity and limitations inherently associated with machine learning in classification methods. Despite several limitations, we aimed to demonstrate that using a combined CNN‐LSTM architecture for advanced EEG signal processing may be a useful approach to distinguish the differential responses of male and female MDD patients to rTMS treatment. The relatively high specificities associated with multivariate autoregressive models and deep learning models indicate that these computational frameworks may provide a basis for an adjunctive therapy.

EEG data before and after rTMS treatment in a population diagnosed with MDD were used in the current study and five EEG‐based deep learning models were created to classify male and female subjects.

## 2 METHODS

### 2.1 Participants

Fifty participants, comprising 25 males and 25 females, received 20 sessions of a bilateral rTMS protocol, that is, low‐frequency (1 Hz) stimulation of the right DLPFC with 120% of the resting motor threshold (RMT) amplitude, in 150 pulse trains each lasting for 10 s with an intertrain interval (ITI) of 2 s. RMT has been defined as the minimum output intensity of a single TMS pulse over the hand motor hotspot that can cause a contraction of the Abductor Pollicis Brevis (APB) muscle after a minimum 5 out of 10 pulses, that can be detected visually (like in our case) or by means of recording electromyography. Stimulation sites corresponding to the right and left DLPFC were located using the EEG 10–20 international system, namely F4 and F3, respectively. Before starting the treatment protocol the included sample in the current study had received a diagnosis of major depression based on a clinical interview by a psychiatrist according to the criteria of the DSM‐V (Regier et al., 2013) and the established exclusion criteria regarding the use of brain stimulation techniques (O'Reardon et al., 2007). For all study participants—divided by sex and treatment response—age, clinical history, and change in Beck Depression Inventory depression scores are presented in Table 1a, specific psychiatric comorbidities in Table 1b, and current medications in Table 1c.

### 2.2 Data collection and EEG recording/preprocessing

EEG data were recorded by a 19‐channel amplifier (Mitsar, Russia) using an ElectroCap (ElectroCap, Inc, OH) on which electrodes were located based on the 10–20 international system. A1+A2 electrodes were used as reference. Impedance was kept below 5 kΩ throughout the experiment and the sampling rate was 500 Hz. Eyes‐open and eyes‐closed resting state EEGs were recorded in an acoustic room for a duration of 10 minutes, before the start and after finishing the rTMS treatment course (20 sessions). In general, EEG artifacts can originate from various physiological or nonphysiological sources such as eye movements, blinks, cardiac or muscle activity, blood pressure, and magnetic field of electronic devices and the power‐line. Artifact‐corrected EEG can be estimated by subtracting pure artifact activity (modeled artifact patterns) from the original artifact‐contaminated EEG. The following preprocessing pipeline was used for removing artifacts without distorting the underlying brain activity.

First, statistically independent (uncorrelated) waveforms and topographies were estimated by the Independent Component Analysis (ICA) method. Therefore, the original signal components resulting from brain activity were identified before artifact removal. A Chebyshev type‐II band‐pass filter was used to eliminate noise from the EEG data. Then, the EEG data set was divided into equal segments with a specific length to guarantee a balanced amount of information in each segment. These segments were then filtered to eliminate noises and interferences. A notch filter at 50 Hz was applied to remove electromagnetic interference of the power‐line/other electronic devices. An Elliptic bandpass filter with cut‐off frequencies of 0.1–60 Hz was used due to its advantages such as requiring less memory and performing with reduced delay time.

The data matrix was made up in the following order: 19 (# of EEG channels) × 50 (# of subjects) × 500 (# of sampling frequency) × 10 (# duration of recording in minutes) × 60 (conversion factor from minutes to seconds for frequency (Hz) × 2 (# of conditions i.e., eyes‐closed and eyes‐open)), and these data frames are directly fed into the model. Before constructing the classification models, patients were categorized into responders or nonresponders to the rTMS treatment, based on the criteria of a minimum reduction of 50% in BDI‐II scores to be considered a responder to the treatment.

### 2.3 Guided selection of deep learning algorithm

**FIGURE 1:**
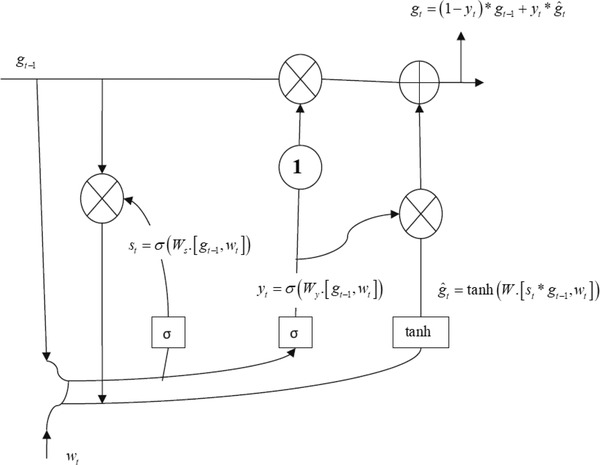
Internal structure of LSTM

Long short‐term memory (LSTM) is derived from the recurrent neural network (RNN), which consists of recurrent structures that locally feed firing strength memories. Thus, RNNs are suitable for time‐series based feature learning tasks, but suffer from gradient vanishing or exploding problems. For this reason, the LSTM network is utilized. In this network, membership functions join the nodes depending on the input variable, and spatial and temporal firing are sustained to assign one‐dimensional membership functions. While learning parameters minimizes the error cost function, the learning structures decide when to generate a rule and activate it with firing strengths exceeding the threshold (Chen et al., 2020; Nguyen et al., 2020; Ouhame et al., 2021). The internal structure of the LSTM algorithm is demonstrated in Figure 1.

The sole use of the LSTM network is not feasible due to considerable memory consumption and computational burden that comes along with it. To overcome this problem, a 1D‐CNN should be added to learn local features on each sensor dimension. 1D convolutional operation on each sensor dimension for the *r*th filter can be expressed in the form of Equation (1):

(1) $$C_{l}^{r}=f^{r}\left(x_{l:l+s-1}^{i}*w^{r}+b^{r}\right),l\in \left\{1,2,\text{…},L-s+1\right\}\text{…}.,$$

where *s* is the filter size, *f*(.) is the activation function, *w* is the weight vector, and *b* is the bias. Output of the 1D convolutional operation can be stated as $C\in R^{(L-s+1)\times 4}$. To obtain a more compact form, a pooling operation can be applied to the output of the 1D convolutional operation. The max‐pooling operation takes the maximum value over that of consecutive features from one sensor dimension. The maximum value is determined by Equation (2):

(2) $$h_{k}^{r}=\max\left(C_{\left(k\right)d+1}^{r},\text{…},C_{\left(k+1\right)d}^{r}\right),k\in \left\{1,2,\text{…},\left[\left(L-s+1\right)/d\right]\right\}..,$$

where *d* is the pooling size and *r* is the number of filters in 1D convolutional operation.

The feature matrix $h\in R^{[(L-s+1)/d]\times r}$ is obtained by performing the 1D convolutional and max‐pooling operations. Since the convolution window slides iteratively from the beginning to the end of the raw signal at each sensor dimension, the first dimension of the feature matrix holds the temporal relationships and the second dimension highlights the high‐level representation explored through the CNN at each sequential step. Outputs of the CNN are high‐level features having temporal characteristics. A flattening layer is used between the convolutional layers and the LSTM layer to reduce the feature maps to a single one‐dimensional vector. Then, the local features are fed into the LSTM to solve the issue of temporal dependency. LSTM provides a solution by saving long‐term memory through memory units that can update the previous hidden state. Output values from the previous CNN layer are fed to gate units. In general, an LSTM network is constituted from a forgetting gate, input gate, input candidate gate, and output gate. The forget gate produces a vector with values between zero and one, which will be then multiplied to the cell state of the former time step for erasing values that were not required and keeping those that are essential for the prediction. The input gate and the input candidate gate work together to render the new cell state, which will be renewed at the next time step. A combined CNN‐LSTM network automatically learns representative features with a high sampling frequency (Abdelhameed et al., 2018; Courtney & Sreenivas, 2020; Nagabushanam et al., 2020; Zhang et al., 2021b).

### 2.4 CNN‐LSTM feature extraction and classification

**FIGURE 2:**
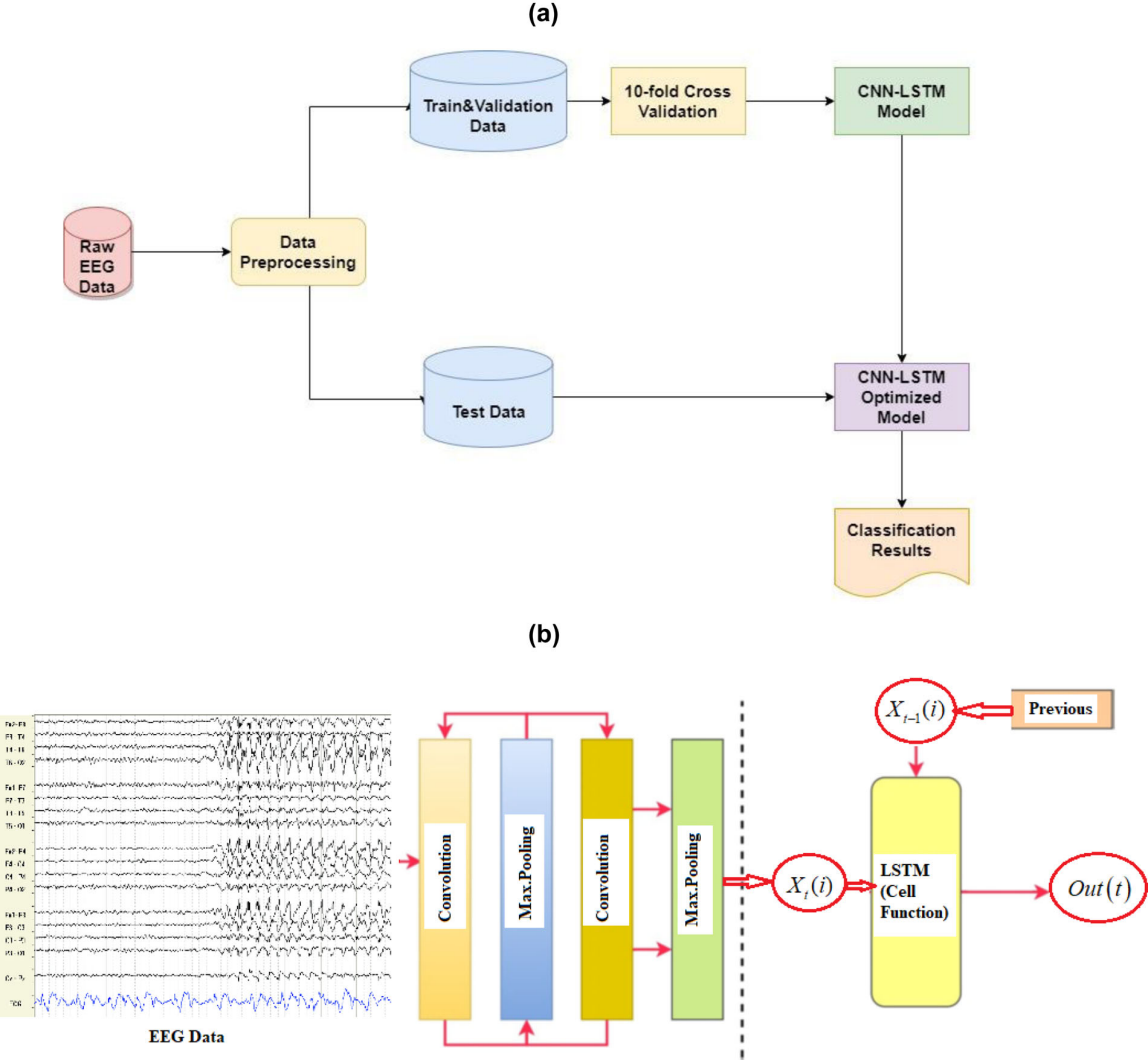
Representation of the proposed deep learning model: (a) block diagram and (b) CNN‐LSTM structure

The algorithm implemented here was based on the publicly available Google Colab, which is a free cloud service that allows AI developers to apply DL‐based algorithms. Our graphic processor unit (GPU) model was a Tesla K80 that supports all Python codes and DL libraries. In our deep learning models, multiple one‐dimensional CNN layers were used to extract features and reduce the length of EEG segments. After that, the LSTM layers were applied to detect sequential relationships to increase the training speed and classification accuracy. While CNN is used to attenuate noise and consider the correlation among multivariable signals associated with each electrode, the LSTM model extracts temporal information and maps time series into separable domains to realize the classification. With the help of this combined model, both the spatial and temporal features can be revealed from the multivariate nonstationary time‐series input data. The block diagram of the proposed model is given in Figure 2.

### 2.5 Training and testing

Five different classification models were built, namely pre‐/post‐TMS female (model 1), pre‐/post‐TMS male (model 2), pre‐TMS female responders versus pre‐TMS female nonresponders (model 3), pre‐TMS male responders versus pre‐TMS male nonresponders (model 4), pre‐TMS responders versus nonresponders across both sexes (model 5). The modeling process was performed using Google Colab via Tensorflow.Keras framework. Training of the classification models was conducted using 10‐fold cross‐validation (CV) for evaluating their robustness against classifying unseen data. In a 10‐fold CV, the data corresponding to each classification model is randomly divided into 10 equivalent folds and class label distributions within each fold. This process was repeated ten times and 90% and 10% of the records are exploited for training and testing, respectively. A new training and testing set was produced by shifting the folds in each iteration in which training records are divided into two subsets, namely a training set and a validation set. The data in each classification model were divided into 10% testing, 70% training, 20% validation set. The models were trained using the training set and optimized by considering the classification results of the validation set.

Due to the small data set size, the data augmentation technique was applied for training to maximize the classification accuracy and ensure minimal overfitting. Generalization performance can be enhanced through the data augmentation method (Lashgari et al., 2020). The data augmentation approach used in this paper is based on the generation of artificial signals in each iteration of the 10‐fold CV by randomly averaging the training set considering about 2% of time instance values in each EEG signal in the train models (Yang et al., 2020; Zhang et al., 2020). Therefore, the training data set was augmented by increasing its size by about %2, for each fold in each model. Given that the total number of the fold is 10, the total number of generated samples after data‐augmentation and partitioning can be found in Table 2.

**TABLE 2:** The size of the partitioned and augmented train and test data per model

	Model 1	Model 2	Model 3	Model 4	Model 5
Initial size of data	19 × 13,841,500	19 × 14,025,000	19 × 6,232,500	19 × 7,179,000	19 × 13,808,000
After reshaping process	27,683 × 500 × 19	28,050 × 500 × 19	12,465 × 500 × 19	14,358 × 500 × 19	27,616 × 500 × 19
After data augmentation	33,220 × 500 × 19	33,660 × 500 × 19	16,620 × 500 × 19	17,230 × 500 × 19	33,140 × 500 × 19
Partitioned into train & validation data	29,898 × 500 × 19	30,294 × 500 × 19	14,958 × 500 × 19	15,507 × 500 × 19	29,826 × 500 × 19
Partitioned into test data	3322 × 500 × 19	3366 × 500 × 19	1662 × 500 × 19	1723 × 500 × 19	3314 × 500 × 19

Recall, precision, specificity, accuracy, f‐measure, kappa value, and ROC‐AUC scores were selected as evaluation metrics for determining the performance of the classification system. These measures were calculated for the testing set of each fold and averaged over the 10‐folds. The hyperparameters were selected as follows: the batch‐size is 128, the optimizer method is adam (adaptive momentum), the evaluation metric is accuracy, and the loss function is cross‐entropy. The baseline 1‐D CNN‐LSTM network architecture used in this paper is presented in Table 3.

**TABLE 3:** Architecture of the proposed model

Layer (type)	Unit type	# Parameters	Output shape
Convolutional (1D)	ReLU	9856	497 × 128
Max Pooling (1D)	–	0	124 × 128
Convolutional (1D)	ReLU	32,832	121 × 64
Max Pooling (1D)	–	0	30 × 64
Convolutional (1D)	ReLU	8224	27 × 32
Max Pooling (1D)	–	0	6 × 32
Convolutional (1D)	ReLU	2064	3 × 16
Convolutional (1D)	ReLU	264	2 × 8
Max pooling (1D)	–	0	1 × 8
LSTM	Tanh	11,800	1 × 50
LSTM	Tanh	7600	1 × 25
LSTM	Tanh	5100	25
Dense (fully connected)	Sigmoid	52	2

## 3 RESULTS

**FIGURE 3:**
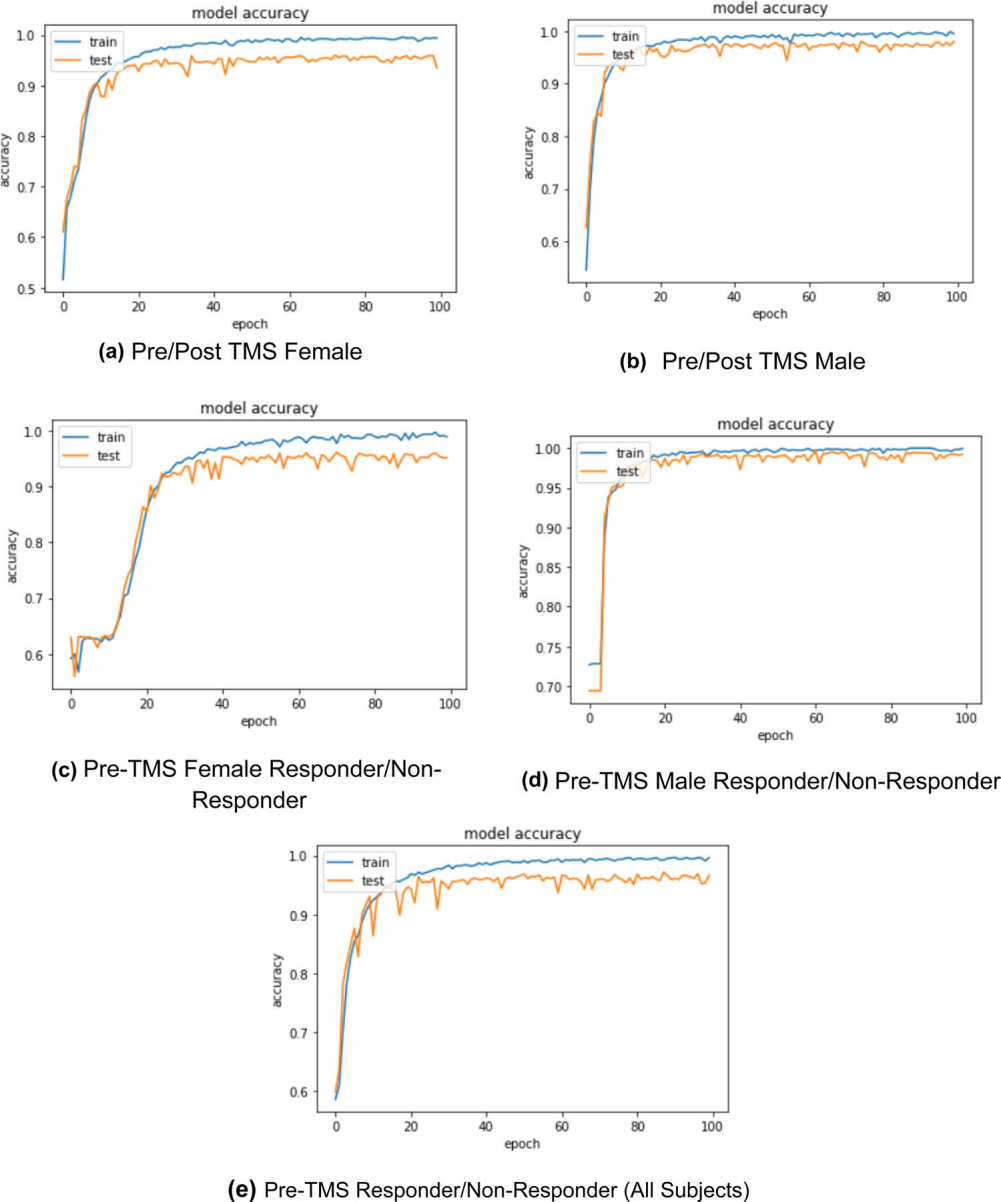
Real‐time accuracy plots for each of the five implemented models

In this study, five different classification models were built. These models include pre‐/post‐TMS female (model 1), pre‐/post‐TMS male (model 2), pre‐TMS female responder versus pre‐TMS female nonresponders (model 3), pre‐TMS male responder versus pre‐TMS male nonresponder (model 4), pre‐TMS responder versus nonresponder for all sexes (model 5). The results of the training process and accuracy plots for the five models that have been implemented in this study are given in Figure 3.

A confusion matrix is a table that is often used to describe the performance of a classifier and visualize the correctly classified labels for each model on a set of test data for which the true values are known. This table layout allows for visualization of the performance of an algorithm and the name stems from the fact that it makes it easy to see whether the system is confusing two classes or not. Each row of the matrix represents the instances in a predicted class, while each column represents the instances in an actual class thus the results were aggregated from the 10 testing results of the 10‐k cross‐validated models.

**FIGURE 4:**
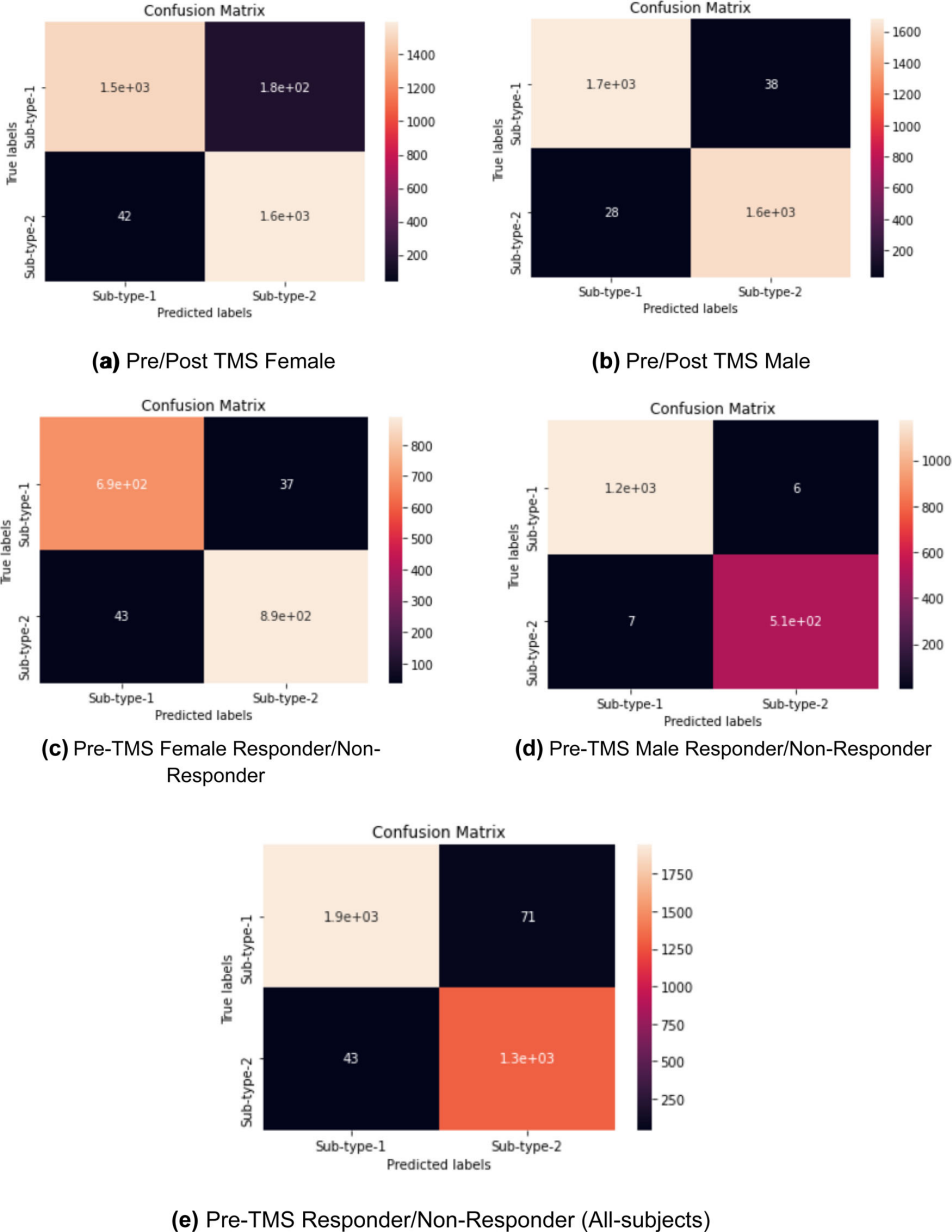
Confusion matrix of the implemented models

A confusion matrix is generated for each model implemented here, as given in Figure 4.

Seen in the confusion matrix for each model, the number of correctly classified labels is high, and its performance is also reflected in its high sensitivity, specificity, overall accuracy, Cohen's kappa value, and AUC values of the models in Table 4.

**TABLE 4:** Comparison of evaluation metrics of the averaged cross‐validated models

	Model 1	Model 2	Model 3	Model 4	Model 5
**Sensitivity**	.973 (±.024)	.984 (±.012)	.941 (±.042)	.994 (±.002)	.978 (±.018)
**Specificity**	.899 (±.022)	0.977 (±0.013)	.960 (±.038)	.988 (±.005)	.948 (±.017)
**Overall Accuracy**	.933 (±.042)	.980 (±.018)	.952 (±.029)	.992 (±.002)	.966 (±.01)
**Cohen's Kappa**	.869 (±.014)	.961 (±.027)	.902 (±.030)	.982 (±.012)	.929 (±.022)
**AUC value**	.944 (±.021)	.944 (±.002)	.953 (±.045)	.992 (±.001)	.973 (±.014)

**TABLE 1a:** Age, clinical history, and change in BDI‐II in study participants

	Total	Male responders	Male nonresponders	Female responders	Female nonresponders
**Sample size**	50	18	7	11	14
**Age (SD)**	34.38 (11.19)	37.77 (12.95)	29.71 (13.02)	32.81 (7.38)	33.57 (10.09)
**Duration of illness in years (SD)**	5.11 (5.12)	5.92 (6.24)	3.5 (3.17)	6.32 (5.47)	3.93 (3.9)
**At least one comorbidity**	35	11	6	8	10
**Neurologic history**	4	1	0	2	1
**At least 1 suicide attempt**	7	1	0	3	2
**BDI pre (SD)**	31.84 (8.04)	30.66 (7.79)	35.42 (9.12)	30.45 (8.46)	32.64 (7.73)
**BDI post (SD)**	14.1 (9.2)	7.94 (5.43)	27.14 (7.98)	8.36 (5.46)	20 (4.94)

*Note*: Beck Depression Inventory (BDI) of which reduction in 50% indicates a response to TMS treatment.

**TABLE 1b:** Frequency of psychiatric comorbidities in study participants

Psychiatric comorbidities	Total (*n* = 50)	Male responders (*n* = 18, 36%)	Male nonresponders (*n* = 7, 14%)	Female responders (*n* = 11, 22%)	Female nonresponders (*n* = 14, 28%)
Adjustment disorder	2	1	None	1	None
Adult ADD	1	1	None	None	None
Cluster B personality disorder	3	1	None	1	1
Cluster C personality disorder	6	2	None	1	2
GAD	17	4	3	5	5
OCD	10	1	4	2	3
Panic disorder	1	None	1	None	None
Phobia	3	None	None	2	1
PTSD	3	None	None	1	2
Social anxiety	1	None	None	None	1

ADD = attention deficit disorder; GAD = generalized anxiety disorder; OCD = obsessive compulsive disorder; PTSD = posttraumatic stress disorder.

**TABLE 1c:** Current medications used in study participants

Classes of medications	Total (*n* = 50)	Male responders (*n* = 18, 36%)	Male nonresponder (*n* = 7, 14%)	Female responders (*n* = 11, 22%)	Female nonresponders (*n* = 14, 28%)
Antipsychotic	23	7	6	6	4
Mood stabilizer	22	7	3	5	7
TCA	15	4	2	5	4
SSRI	13	6	2	3	2
Benzodiazepine	8	3	1	1	3
SNRI	3	1	None	2	None
Beta‐blockers	3	None	None	1	2
NDRI	2	1	None	None	1
SARI	2	None	None	2	None
Anxiolytic	1	None	1	None	None
Antidepressants	1	None	None	1	None
Sedative‐hypnotics	1	None	None	None	1
Hormones (melatonin)	1	1	None	None	None

TCA = tricyclic antidepressants; SSRI = selective serotonin reuptake inhibitor; SNRI = serotonin–norepinephrine reuptake inhibitor; NDRI = norepinephrine‐dopamine reuptake inhibitor; SARI = serotonin antagonist and reuptake inhibitors.

The results for each model are promising for employment in a clinical context; however, a well‐defined graphical user interface (GUI) needs to be developed to make the process more user‐friendly in addition to further validations with more data for better approximation and generalization. Each of the five models performed with high sensitivity, specificity, and overall accuracy thus resulting in a high‐classification performance. Cohen's kappa is a robust statistic useful for the purposes of either interrater or intrarater reliability testing. Similar correlation coefficients range from −1 to +1, where 0 represents the degree of agreement that can be expected from random chance, and 1 represents perfect agreement between raters. The kappa values for the proposed models all have high areas under the ROC curve.

## 4 DISCUSSION

Although all the five implemented models in the current study were able to classify the pre‐ and post‐rTMS responses with an accuracy of above 91%, model 4 performed significantly better in classifying responsivity in pre‐rTMS data in males. However, this does not necessarily suggest that males respond better to rTMS, but only highlights the fact that model 4 outperformed the others in determining sex differences in response to rTMS. Several factors may have contributed to this result, one of them being that out of 25 participants of each sex, there were 14 female while only 7 male nonresponders. This suggests that the model may not have been able to classify the females with the same accuracy as the males. The small sample size—as small as 7 in the male responder group—poses an additional limitation on the significance of the results reported. Since in this study we did not apply statistical methods, it is not correct to express the findings in terms of statistical significance. Instead we used other metrics specific to machine learning algorithms such as the confusion matrix of the feature set, area under ROC curve, and kappa values which are listed in Figure 4 and Table 4.

According to a recent review of sex differences in response to neuromodulation treatments Phillips et al. (2020) have noted that differences in cortical thickness may require different protocol parameters such as higher intensities to achieve comparable results in females. Considering that the treatment in the current work included low‐ and high‐frequency stimulation of right and left DLPFC, respectively, further investigation of the implications of other rTMS parameters regarding treatment response, also as a function of sex, is required to realize a more personalized treatment approach. It must also be noted that the study is done on an Iranian sample of depressed patients; therefore, cultural factors of MDD should have been accounted for to be able to make more firm conclusions based on the reported results. It has been noted in a recent epidemiological review that the prevalence of MDD is twice as high for women in Iran and some neighboring countries (Gharraee et al., 2019) thus the cultural and geographical context in relation to sex may have affected the result tendencies and thus must be considered in future investigations to understand sex differences in treatment response to rTMS.

It is worth noting that model 5 was able to predict response to rTMS with an accuracy of 94% regardless of sex, using the baseline's pre‐rTMS EEG. Thus, the results of the current investigation provides that it is feasible to make preliminary predictions about the likelihood of the efficacy of rTMS for MDD patients, given all other variables are standardized.

## 5 CONCLUSIONS

To the best of our knowledge, this is the first investigation using deep learning methods to specifically investigate sex differences in response to rTMS based on EEG in the context of MDD. The proposed method is robust and automated due to the representation power of time‐invariant features from raw EEG signals (Liao et al., 2021). In this methodology, intraslice features of brain images are processed and extracted and can be adopted for other physiological data and imaging modalities such as fMRI, CT, or PET (Cheng & Liu, 2017). The results verify that the proposed method could achieve comparable performance with other state‐of‐the‐art methods to identify sex differences in response to rTMS in MDD patients. Therefore, it is safe to believe that our developed model can also be used for the purposes of accurate diagnosis of other neurological and psychiatric pathologies as well (Ay et al., 2019; Saeedi et al., 2021).

While advances in neuroimaging techniques have rapidly improved over the past few decades, there is still a lack of acceptable precision for the diagnosis and treatment of mental illness (Etkin, 2019). The paradigm shift to a personalized approach toward treatment in recent years has led to the use of techniques such as deep learning among other computational methods applied on neurobiological data, to enhance diagnosis accuracy and validity (de Bardeci et al., 2021). The use of neural networks may lead to a more effective and efficient way to identify previously unrecognized patterns in EEG traces by reducing the need to extract diagnostic information from highly complex EEG time‐series and shifting to a more automated, objective, and reliable approach. Physician's management of psychiatric illness can thus be facilitated with the help of computational techniques in psychiatry (de Bardeci et al., 2021).

The preliminary results of the models can have extensive implications in providing precise treatment parameters or target brain regions for innovative neuromodulation treatments especially for vulnerable populations such as females with MDD. There is definitely the need for further studies or meta‐analyses of studies on sex as well as cultural differences in diagnosis of mental illness and response to specific treatments. Considering the relative ease of use and accessibility of EEG equipment, it is a very promising candidate for investigations of predictors of response to treatment and diagnostic markers. With further replicable deep learning investigations on EEG data across diverse populations, mental health care providers can will be able to provide more efficacious treatment protocols, personalized to a patient's sex, medication profile, or transdiagnostic symptoms, among many other potential variables each of which requiring more research efforts and attention.

### PEER REVIEW

The peer review history for this article is available at https://publons.com/publon/10.1002/brb3.2696

## Data Availability

The data that support the findings of this study are available from the corresponding author upon reasonable request.

## References

[brb32696-bib-0001] Abbasi, B. , & Goldenholz, D. M. (2019). Machine learning applications in epilepsy. Epilepsia, 60(10), 2037–2047. 10.1111/epi.16333 31478577 PMC9897263

[brb32696-bib-0002] Abdelhameed, A. M. , Daoud, H. G. , & Bayoumi, M. (2018). Deep convolutional bidirectional LSTM recurrent neural network for epileptic seizure detection. Presented at the 2018 16th IEEE International New Circuits and Systems Conference (NEWCAS), Montreal, QC. 10.1109/newcas.2018.8585542

[brb32696-bib-0003] Ay, B. , Yildirim, O. , Talo, M. , Baloglu, U. B. , Aydin, G. , Puthankattil, S. D. , & Acharya, U. R. (2019). Automated depression detection using deep representation and sequence learning with EEG signals. Journal of Medical Systems, 43(7), 205. 10.1007/s10916-019-1345-y 31139932

[brb32696-bib-0004] Bares, M. , Novak, T. , Kopecek, M. , Brunovsky, M. , Stopkova, P. , & Höschl, C. (2015). The effectiveness of prefrontal theta cordance and early reduction of depressive symptoms in the prediction of antidepressant treatment outcome in patients with resistant depression: Analysis of naturalistic data. European Archives of Psychiatry and Clinical Neuroscience, 265(1), 73–82. 10.1007/s00406-014-0506-8 24848366

[brb32696-bib-0005] Cheng, D. , & Liu, M. (2017). Combining convolutional and recurrent neural networks for Alzheimer's disease diagnosis using PET images. *2017 IEEE International Conference on Imaging Systems and Techniques (IST)*. Presented at the 2017 IEEE International Conference on Imaging Systems and Techniques (IST), Beijing. 10.1109/ist.2017.8261461

[brb32696-bib-0006] Chen, Z. , Wu, M. , Cui, W. , Liu, C. , & Li, X. (2020). An attention based CNN‐LSTM approach for sleep‐wake detection with heterogeneous sensors. IEEE Journal of Biomedical and Health Informatics, 10.1109/JBHI.2020.3006145 32749983

[brb32696-bib-0007] Cosmo, C. , Zandvakili, A. , Petrosino, N. J. , Berlow, Y. A. , & Philip, N. S. (2021). Repetitive transcranial magnetic stimulation for treatment‐resistant depression: Recent critical advances in patient care. Current Treatment Options in Psychiatry, 8, 47–63. 10.1007/s40501-021-00238-y 33723500 PMC7946620

[brb32696-bib-0008] Courtney, L. , & Sreenivas, R. (2020). Using deep convolutional LSTM networks for learning spatiotemporal features. In Lecture notes in computer science. lecture notes in computer science (pp. 307–320). Cham: Springer International Publishing. 10.1007/978-3-030-41299-9_24

[brb32696-bib-0009] Craik, A. , He, Y. , & Contreras‐Vidal, J. L. (2019). Deep learning for electroencephalogram (EEG) classification tasks: A review. Journal of Neural Engineering, 16(3), 031001. 10.1088/1741-2552/ab0ab5 30808014

[brb32696-bib-0010] de Aguiar Neto, F. S. , & Rosa, J. L. G. (2019). Depression biomarkers using non‐invasive EEG: A review. Neuroscience and Biobehavioral Reviews, 105, 83–93. 10.1016/j.neubiorev.2019.07.021 31400570

[brb32696-bib-0011] de Bardeci, M. , Ip, C. T. , & Olbrich, S. (2021). Deep learning applied to electroencephalogram data in mental disorders: A systematic review. Biological Psychology, 162, 108117. 10.1016/j.biopsycho.2021.108117 33991592

[brb32696-bib-0012] de la Salle, S. , Jaworska, N. , Blier, P. , Smith, D. , & Knott, V. (2020). Using prefrontal and midline right frontal EEG‐derived theta cordance and depressive symptoms to predict the differential response or remission to antidepressant treatment in major depressive disorder. Psychiatry Research. Neuroimaging, 302, 111109. 10.1016/j.pscychresns.2020.111109 32480044 PMC10773969

[brb32696-bib-0013] Downar, J. , & Daskalakis, Z. J. (2013). New targets for rTMS in depression: A review of convergent evidence. Brain Stimulation, 6(3), 231–240. 10.1016/j.brs.2012.08.006 22975030

[brb32696-bib-0014] Etkin, A. (2019). A Reckoning and research agenda for neuroimaging in psychiatry. The American Journal of Psychiatry, 176(7), 507–511. 10.1176/appi.ajp.2019.19050521 31256624

[brb32696-bib-0015] Gharraee, B. , Zahedi Tajrishi, K. , Sheybani, F. , Tahmasbi, N. , Mirzaei, M. , Farahani, H. , & Naserbakht, M. (2019). Prevalence of major depressive disorder in the general population of Iran: A systematic review and meta‐analysis. Medical Journal of the Islamic Republic of Iran, 33, 151. 10.34171/mjiri.33.151 32280657 PMC7137832

[brb32696-bib-0016] Greenberg, B. D. , Philip, N. S. , Fortin‐Ashburne, K. , & Carpenter, L. L. (2021). The COBRE Center for Neuromodulation (CCN) at Butler Hospital: Clinical‐translational research in human brain stimulation. Rhode Island Medical Journal, 104(2), 30–33. Retrieved from https://www.ncbi.nlm.nih.gov/pubmed/33648316 PMC8211205 33648316

[brb32696-bib-0017] Huang, C.‐C. , Wei, I.‐H. , Chou, Y.‐H. , & Su, T.‐P. (2008). Effect of age, gender, menopausal status, and ovarian hormonal level on rTMS in treatment‐resistant depression. Psychoneuroendocrinology, 33(6), 821–831. 10.1016/j.psyneuen.2008.03.006 18468810

[brb32696-bib-0018] Hu, Y.‐T. , Hu, X.‐W. , Han, J.‐F. , Zhang, J.‐F. , Wang, Y.‐Y. , Wolff, A. , Tremblay, S. , Tan, Z.‐L. , & Northoff, G. (2021). Childhood trauma mediates repetitive transcranial magnetic stimulation efficacy in major depressive disorder. European Archives of Psychiatry and Clinical Neuroscience, 271, 1255–1263. 10.1007/s00406-021-01279-3 34117915

[brb32696-bib-0019] Kessler, R. C. , Berglund, P. , Demler, O. , Jin, R. , Koretz, D. , Merikangas, K. R. , Rush, A. J. , Walters, E. E. , & Wang, P. S. (2003). The epidemiology of major depressive disorder: Results from the national comorbidity survey replication (NCS‐R). JAMA: The Journal of the American Medical Association, 289(23), 3095–3105. 10.1001/jama.289.23.3095 12813115

[brb32696-bib-0020] Lashgari, E. , Liang, D. , & Maoz, U. (2020). Data augmentation for deep‐learning‐based electroencephalography. Journal of Neuroscience Methods, 346, 108885. 10.1016/j.jneumeth.2020.108885 32745492

[brb32696-bib-0021] Leuchter, A. F. , Cook, I. A. , Marangell, L. B. , Gilmer, W. S. , Burgoyne, K. S. , Howland, R. H. , Trivedi, M. H. , Zisook, S. , Jain, R. , McCracken, J. T. , Fava, M. , Iosifescu, D. , & Greenwald, S. (2009). Comparative effectiveness of biomarkers and clinical indicators for predicting outcomes of SSRI treatment in Major Depressive Disorder: Results of the BRITE‐MD study. Psychiatry Research, 169(2), 124–131. 10.1016/j.psychres.2009.06.004 19712979

[brb32696-bib-0022] Liao, J. , Liu, D. , Su, G. , & Liu, L. (2021). Recognizing diseases with multivariate physiological signals by a DeepCNN‐LSTM network. Applied Intelligence, 51, 7933–7945. 10.1007/s10489-021-02309-2

[brb32696-bib-0023] Nagabushanam, P. , Thomas George, S. , & Radha, S. (2020). EEG signal classification using LSTM and improved neural network algorithms. Soft Computing, 24(13), 9981–10003. 10.1007/s00500-019-04515-0

[brb32696-bib-0024] Nguyen, N. T. , Tran, D. Q. , Nguyen, N. T. , & Nguyen, H. Q. (2020). A CNN‐LSTM architecture for detection of intracranial hemorrhage on CT scans. *medRxiv*, 2020.04.17.20070193. 10.1101/2020.04.17.20070193

[brb32696-bib-0025] O'Reardon, J. P. , Solvason, H. B. , Janicak, P. G. , Sampson, S. , Isenberg, K. E. , Nahas, Z. , McDonald, W. M. , Avery, D. , Fitzgerald, P. B. , Loo, C. , Demitrack, M. A. , George, M. S. , & Sackeim, H. A. (2007). Efficacy and safety of transcranial magnetic stimulation in the acute treatment of major depression: A multisite randomized controlled trial. Biological Psychiatry, 62(11), 1208–1216. 10.1016/j.biopsych.2007.01.018 17573044

[brb32696-bib-0026] Ouhame, S. , Hadi, Y. , & Ullah, A. (2021). An efficient forecasting approach for resource utilization in cloud data center using CNN‐LSTM model. Neural Computing & Applications, 33, 10043–10055. 10.1007/s00521-021-05770-9

[brb32696-bib-0027] Perugi, G. , Musetti, L. , Simonini, E. , Piagentini, F. , Cassano, G. B. , & Akiskal, H. S. (1990). Gender‐mediated clinical features of depressive illness. The importance of temperamental differences. The British Journal of Psychiatry: The Journal of Mental Science, 157, 835–841. 10.1192/bjp.157.6.835 2289093

[brb32696-bib-0028] Philip, N. S. , Barredo, J. , Aiken, E. , & Carpenter, L. L. (2018). Neuroimaging mechanisms of therapeutic transcranial magnetic stimulation for major depressive disorder. Biological Psychiatry. Cognitive Neuroscience and Neuroimaging, 3(3), 211–222. 10.1016/j.bpsc.2017.10.007 29486862 PMC5856477

[brb32696-bib-0029] Phillips, A. , Sami, S. , & Adamson, M. (2020). Sex differences in neuromodulation treatment approaches for traumatic brain injury: A scoping review. The Journal of Head Trauma Rehabilitation, 35(6), 412–429. 10.1097/HTR.0000000000000631 33165154

[brb32696-bib-0030] Picco, L. , Subramaniam, M. , Abdin, E. , Vaingankar, J. A. , & Chong, S. A. (2017). Gender differences in major depressive disorder: Findings from the Singapore Mental Health Study. Singapore Medical Journal, 58(11), 649–655. 10.11622/smedj.2016144 27526704 PMC5691228

[brb32696-bib-0031] Regier, D. A. , Kuhl, E. A. , & Kupfer, D. J. (2013). The DSM‐5: Classification and criteria changes. World Psychiatry: Official Journal of the World Psychiatric Association, 12(2), 92–98. 10.1002/wps.20050 PMC3683251 23737408

[brb32696-bib-0032] Rogers, L. M. , & Dhaher, Y. Y. (2017). Female sex hormones modulate the response to low‐frequency rTMS in the human motor cortex. Brain Stimulation, 10(4), 850–852. 10.1016/j.brs.2017.02.010 28330632

[brb32696-bib-0033] Rubinow, D. R. , & Schmidt, P. J. (2019). Sex differences and the neurobiology of affective disorders. Neuropsychopharmacology: Official Publication of the American College of Neuropsychopharmacology, 44(1), 111–128. 10.1038/s41386-018-0148-z 30061743 PMC6235863

[brb32696-bib-0034] Saeedi, A. , Saeedi, M. , Maghsoudi, A. , & Shalbaf, A. (2021). Major depressive disorder diagnosis based on effective connectivity in EEG signals: A convolutional neural network and long short‐term memory approach. Cognitive Neurodynamics, 15(2), 239–252. 10.1007/s11571-020-09619-0 33854642 PMC7969675

[brb32696-bib-0035] Seney, M. L. , Huo, Z. , Cahill, K. , French, L. , Puralewski, R. , Zhang, J. , Logan, R. W. , Tseng, G. , Lewis, D. A. , & Sibille, E. (2018). Opposite molecular signatures of depression in men and women. Biological Psychiatry, 84(1), 18–27. 10.1016/j.biopsych.2018.01.017 29548746 PMC6014892

[brb32696-bib-0036] Tremblay, S. , Rogasch, N. C. , Premoli, I. , Blumberger, D. M. , Casarotto, S. , Chen, R. , Lazzaro, V. D. , Farzan, F. , Ferrarelli, F. , Fitzgerald, P. B. , Hui, J. , Ilmoniemi, R. J. , Kimiskidis, V. K. , Kugiumtzis, D. , Lioumis, P. , Pascual‐Leone, A. , Pellicciari, M. C. , Rajji, T. , Thut, G. , … Daskalakis, Z. J. (2019). Clinical utility and prospective of TMS‐EEG. Clinical Neurophysiology: Official Journal of the International Federation of Clinical Neurophysiology, 130(5), 802–844. 10.1016/j.clinph.2019.01.001 30772238

[brb32696-bib-0037] Tripp, A. , Oh, H. , Guilloux, J.‐P. , Martinowich, K. , Lewis, D. A. , & Sibille, E. (2012). Brain‐derived neurotrophic factor signaling and subgenual anterior cingulate cortex dysfunction in major depressive disorder. The American Journal of Psychiatry, 169(11), 1194–1202. 10.1176/appi.ajp.2012.12020248 23128924 PMC3638149

[brb32696-bib-0038] Williams, L. M. , Coman, J. T. , Stetz, P. C. , Walker, N. C. , Kozel, F. A. , George, M. S. , Yoon, J. , Hack, L. M. , Madore, M. R. , Lim, K. O. , Philip, N. S. , & Holtzheimer, P. E. (2021). Identifying response and predictive biomarkers for Transcranial magnetic stimulation outcomes: Protocol and rationale for a mechanistic study of functional neuroimaging and behavioral biomarkers in veterans with Pharmacoresistant depression. BMC Psychiatry, 21(1), 35. 10.1186/s12888-020-03030-z 33435926 PMC7805238

[brb32696-bib-0039] Yang, T. , Lee, S. , & Lee, T. (2020). Detection of drivers’ anxiety invoked by driving situations using multimodal biosignals. Processes, 8, 155 10.3390/pr8020155

[brb32696-bib-0040] Yao, L. , Zhao, X. , Xu, Z. , Chen, Y. , Liu, L. , Feng, Q. , & Chen, F. (2020). Influencing factors and machine learning‐based prediction of side effects in psychotherapy. Frontiers in Psychiatry /Frontiers Research Foundation, 11, 537442. 10.3389/fpsyt.2020.537442 PMC7744296 33343404

[brb32696-bib-0041] Zhang, J. , Tang, Z. , Gao, J. , Lin, L. , Liu, Z. , Wu, H. , Liu, F. , & Yao, R. (2021a). Automatic detection of obstructive sleep apnea events using a deep CNN‐LSTM model. Computational Intelligence and Neuroscience, 2021, 5594733. 10.1155/2021/5594733 33859679 PMC8009718

[brb32696-bib-0042] Zhang, Y. , Guo, Y. , Yang, P. , Chen, W. , & Lo, B. (2020). Epilepsy seizure prediction on EEG using common spatial pattern and convolutional neural network. IEEE Journal of Biomedical and Health Informatics, 24(2), 465–474. 10.1109/jbhi.2019.2933046 31395568

[brb32696-bib-0043] Zhang, Y. , Wu, W. , Toll, R. T. , Naparstek, S. , Maron‐Katz, A. , Watts, M. , Gordon, J. , Jeong, J. , Astolfi, L. , Shpigel, E. , Longwell, P. , Sarhadi, K. , El‐Said, D. , Li, Y. , Cooper, C. , Chin‐Fatt, C. , Arns, M. , Goodkind, M. S. , Trivedi, M. H. , … Etkin, A. (2021b). Identification of psychiatric disorder subtypes from functional connectivity patterns in resting‐state electroencephalography. Nature Biomedical Engineering, 5(4), 309–323. 10.1038/s41551-020-00614-8 PMC8053667 33077939

